# Supplementary material for: The Use of COVID-19 Mobile Apps in Connecting Patients with Primary Healthcare in 30 Countries: Eurodata Study
Source: Healthcare (Basel). 2024 Jul 16;12(14):1420. doi: 10.3390/healthcare12141420 (PMC11275920; doi:10.3390/healthcare12141420)
Supplement: Supplementary file 1 [file healthcare-12-01420-s001.zip › healthcare-3047367-supplementary.pdf]

Supplementary files:

Supplementary file S1: Non-EU countries (and territories) that have joined the EU Digital COVID Certificate system

Supplementary file S2: Definitions of each service and healthcare professional in this study

Supplementary file S3: Sources of the COVID-19 Apps in each country.

Suplementary file S4: STROBE Statement

Supplementary file S1: Non-EU countries (and territories) that have joined the EU Digital COVID Certificate system

1. Albania	21. Lebanon	41. Taiwan
2. Andorra	22. Liechtenstein	42. Thailand
3. Armenia	23. Madagascar	43. Tunisia
4. Azerbaijan	24. Malaysia	44. Togo
5. Bahrain	25. Moldova	45. Türkiye
6. Benin	26. Monaco	46. Ukraine
7. Brazil	27. Montenegro	47. United Arab Emirates
8. Cabo Verde	28. Morocco	48. United Kingdom and the Crown Dependencies (Jersey, Guernsey and the Isle of Man)
9. Colombia	29. New Zealand	49. Uruguay
10. Ecuador	30. North Macedonia	50. Vietnam
11. El Salvador	31. Norway	51. The Vatican
12. Faroe Islands	32. Oman	
13. Georgia	33. Panama	
14. Hong Kong	34. Peru	
15. Israel	35. Philippines	
16. Iceland	36. San Marino	
17. Indonesia	37. Serbia	
18. Jordan	38. Seychelles	
19. Republic of Korea	39. Singapore	
20. Kosovo	40. Switzerland	

Supplementary file S2: Definitions of each service and healthcare professional in this study. The definitions have been adapted to the COVID-19 pandemic from the MeSH terms in PubMed:

- **A&E:** Accident & Emergency department or Emergency Department. Hospital department responsible for the administration and provision of immediate medical to the COVID-19 patient.
- **Contact tracing:** Identification of those persons who have had such an association with an infected person, animal, or contaminated environment as to have had the opportunity to acquire the infection.
- **COVID-19 Telephone Hotline:** A direct communication system, usually telephone, established for instant contact. It is designed to provide only information about COVID-19 and assistance through trained personnel and is used for counselling and referrals.
- **Digital COVID-19 certificate or EU Digital COVID-19 certificate:** a digital proof that a person has either: been vaccinated against COVID-19 or received a negative test result or recovered from COVID-19
- **E-Health/M-Health:** Delivery of health services via remote telecommunications. This includes interactive consultative and diagnostic services.
- **GP:** General Practitioner or family doctor. Doctors who are responsible for the provision of comprehensive and continuing care to every individual seeking medical care irrespective of age, sex and illness and they care for individuals in the context of their community (WONCA Europe definition: https://www.woncaeurope.org/page/definition-of-general-practice-family-medicine)
- **Health Information Interoperability:** Automatic and seamless exchange or cross-talk across HEALTH INFORMATION SYSTEM
- **Mobile Applications/ apps:** Computer programs or software installed on mobile electronic devices which support a wide range of functions and uses which include television, telephone, video, music, word processing, and Internet service.
- **Online portal:** A secure online website that provides patients convenient 24-hour access to personal health information via an Internet connection
- **Primary health care:** Care which provides integrated, accessible health care services provided by a GP or a primary care nurse in the context of family and community
- **Public Health:** Public health refers to all organized measures (whether public or private) to prevent disease, promote health, and prolong life among the population as a whole. Its activities aim to provide conditions in which people can be healthy and focus on entire populations, not on individual patients or diseases. Thus, public health is concerned with the total system and not only the eradication of a particular disease (WHO definition: https://www.euro.who.int/__data/assets/pdf_file/0007/152683/e95877.pdf)

- **Social Services:** The use of community resources, individual case work, or group work to promote the adaptive capacities of individuals in relation to their social and economic environments.
- **112:** Phone line for emergency in many European countries.
- **113:** Phone line for ambulance and/or emergency medicine in Latvia

Supplementary file S3: Sources of the COVID-19 Apps in each country, accessed 1st of March 2023.

COUNTRY	Sources of information
**Austria**	https://www.stopp-corona.at<br>https://greenpassapp.eu/<br>https://www.sozialministerium.at/Corona/allgemeine-informationen/gruener-pass.html#der-gruene-pass
**Belarus**	https://minzdrav.gov.by/ru/dlya-belorusskikh-grazhdan/COVID-19/informatsiya-o-vydache-sertifikata-o-vaktsinatsii.php
**Belgium**	https://coronalert.be/en/<br>www.mijngezondheid.be<br>https://www.info-coronavirus.be/en/self-test/<br>https://www.myhealth.belgium.be/#/<br>https://covidsafe.be/en
**Bulgaria**	https://virusafe.info<br>https://play.google.com/store/apps/details?id=dcc.check.bg&hl=es_419&gl=US
**Croatia**	https://www.koronavirus.hr/stop-covid-19-723/723<br>https://www.koronavirus.hr/uploads/Stop_COVID_19_Data_Protection_Impact_Assesment_Summary_2020_11_16_58dea76816.pdf<br>https://play.google.com/store/apps/details?id=hr.akd.dzp&hl=en_US&gl=US<br>https://www.eudigitalnacovidpotvrda.hr/en
**Czech Republic**	https://erouska.cz<br>https://erouska.cz/en/caste-dotazy
**Cyprus**	https://covtracer.dmrid.gov.cy/dmrid/covtracer/covtracer.nsf/home_el/home_el?opendocument<br>https://www.pio.gov.cy/coronavirus/uploads/11032021_pressconfYfypourgouapplicationENG.pdf<br>https://www.pio.gov.cy/coronavirus/eng/categories/press-releases<br>https://apps.apple.com/us/app/covpass-cyprus/id1572945485

**Finland**	https://thl.fi/en/web/thlfi-en/-/latest-version-of-the-koronavilkku-app-was-released-17-june-with-instructions-for-vaccinated-people-who-receive-an-exposure-notification<br>https://thl.fi/en/web/thlfi-en/-/koronavilkku-application-has-been-shut-down-thanks-to-all-users-of-the-application-<br>https://www.solita.fi/en/customers/koronavilkku-the-worlds-most-downloaded-coronavirus-app/<br>https://www.omaolo.fi/usein-kysyttya<br>https://www.omaolo.fi/kayttoohjeet/omaolo-instructions.pdf<br>https://www.kanta.fi/en/my-kanta-pages<br>https://digifinland.fi/en/our-operations/omaolo-service/
**France**	https://www.economie.gouv.fr/tousanticovid<br>https://tousanticovid.stonly.com/kb/fr
**Germany**	https://www.bundesregierung.de/breg-de/themen/corona-warn-app/corona-warn-app-englisch<br>https://www.bundesregierung.de/breg-de/themen/corona-warn-app/corona-warn-app-englisch/corona-warn-app-faq-1758636<br>https://play.google.com/store/apps/details?id=de.rki.covpass.app&hl=es&gl=US
**Greece**	https://play.google.com/store/apps/details?id=gr.gov.dcc.wallet&hl=en&gl=US
**Hungary**	https://nextsense.com/contact-tracing-technology.nspx<br>https://e-egeszsegugy.gov.hu/web/eeszt-information-portal<br>https://e-egeszsegugy.gov.hu/web/eeszt-information-portal/data-protection
**Ireland**	https://www.covidtracker.ie<br>https://www2.hse.ie/conditions/coronavirus/covid-tracker-app/why-use-the-covid-tracker-app.html
**Israel**	https://govextra.gov.il/ministry-of-health/hamagen-app/download-en/
**Italy**	https://www.immuni.italia.it/<br>https://io.italia.it/certificato-verde-green-pass-covid/#:~:text=Il%20tuo%20certificato%20COVID%20su%20IO%20app&text=Su%20IO%20riceverai%20un%20messaggio,app%20con%20SPID%20o%20CIE<br>https://sinfonia.regione.campania.it/preview/ecovid
**Latvia**	https://www.apturicovid.lv/#en<br>https://covid19sertifikats.lv<br>https://covid19.gov.lv/index.php/en/covid-19/about-covid-19/digital-covid-19-certificate
**Lithuania**	https://koronastop.lrv.lt/en/<br>https://koronastop.lrv.lt/en/faq/<br>https://sam.lrv.lt/uploads/sam/documents/files/angliškas.pdf<br>https://www.esveikata.lt
**Luxembourg**	https://guichet.public.lu/en/citoyens/sante-social/coronavirus/covidcheck/demande-certificat-europeen-vaccination-retablissement.html
**North of Macedonia**	https://mhealth-hub.org/stopkorona<br>https://vakcinacija.mk/en/landing

**Poland**	https://www.gov.pl/web/stopcovid-en<br>https://www.gov.pl/web/certificate/about-certyficate
**Portugal**	https://stayawaycovid.pt/pt/<br>https://mhealth-hub.org/stayaway-covid<br>https://eportugal.gov.pt/en/servicos/pedir-o-certificado-digital-covid-da-ue<br>https://apps.apple.com/us/app/sns-24/id1192353854<br>http://www.spms.min-saude.pt/2019/05/trace-covid-19/<br>https://mhealth-hub.org/stayaway-covid<br>https://www.sns24.gov.pt/guia/app-sns-24/#quais-os-servicos-que-estao-disponiveis-nesta-app
**Romania**	https://www.adr.gov.ro/diasporahub-ro/<br>https://vaccinare-covid.gov.ro/rovaccinare/<br>https;//certificat-covid.gov.ro<br>https>// vaccinare-covid.ro
**Serbia**	https://cep.org.rs/wp-content/uploads/2023/01/COVID-19-tracing-app-in-Serbia.pdf<br>https://www.e-zdravlje.gov.rs/landing/?v=20230105
**Slovenia**	https://www.gov.si/en/topics/coronavirus-disease-covid-19/the-ostanizdrav-mobile-application/<br>https://podatki.gov.si/dataset/statisticni-podatki-aplikacije-ostanizdrav/<br>https://play.google.com/store/apps/details?id=si.gov.ostanizdrav&hl=es&gl=US<br>https://covid-19.sledilnik.org/en/about<br>https://covid-19.sledilnik.org/en/stats<br>Zvem.ezdrav.si
**Spain**	https://radarcovid.gob.es/home
**Sweden**	https://www.ehalsomyndigheten.se/languages/english/covid-certificate/
**Turkey**	https://hayatevesigar.saglik.gov.tr<br>https://hayatevesigar.saglik.gov.tr/HES.pdf<br>https://apps.apple.com/es/app/hayat-eve-sığar/id1505756398
**United Kingdom**	https://www.gov.uk/government/collections/nhs-covid-19-app
**Ukraine**	https://diia.gov.ua/

Suplementary file S4

STROBE Statement—Checklist : **The Use of COVID-19 Mobile Apps in Connecting Patients with Primary Health Care in 30 countries: Eurodata study.**

	Item No	Recommendation
**Title and abstract**	1-4	(*a*) Indicate the study's design with a commonly used term in the title or the abstract
		(*b*) Provide in the abstract an informative and balanced summary of what was done and what was found
**Introduction**		
Background/rationale	5	Explain the scientific background and rationale for the investigation being reported
Objectives	6	State specific objectives, including any prespecified hypotheses
**Methods**		
Study design	6	Present key elements of study design early in the paper
Setting	6	Describe the setting, locations, and relevant dates, including periods of recruitment, exposure, follow-up, and data collection
Participants	6	(*a*) Give the eligibility criteria, and the sources and methods of selection of participants
Variables	7	Clearly define all outcomes, exposures, predictors, potential confounders, and effect modifiers. Give diagnostic criteria, if applicable
Data sources/ measurement	7	For each variable of interest, give sources of data and details of methods of assessment (measurement). Describe comparability of assessment methods if there is more than one group
Bias	-	Describe any efforts to address potential sources of bias
Study size	-	Explain how the study size was arrived at
Quantitative variables	-	Explain how quantitative variables were handled in the analyses. If applicable, describe which groupings were chosen and why
Statistical methods	7-8	(*a*) Describe all statistical methods, including those used to control for confounding
		(*b*) Describe any methods used to examine subgroups and interactions
		(*c*) Explain how missing data were addressed
		(*d*) If applicable, describe analytical methods taking account of sampling strategy
		(*e*) Describe any sensitivity analyses
**Results**		
Participants	-	(a) Report numbers of individuals at each stage of study—eg numbers potentially eligible, examined for eligibility, confirmed eligible, included in the study, completing follow-up, and analysed
		(b) Give reasons for non-participation at each stage

		(c) Consider use of a flow diagram
Descriptive data	8-9	(a) Give characteristics of study participants (eg demographic, clinical, social) and information on exposures and potential confounders
		(b) Indicate number of participants with missing data for each variable of interest
Outcome data	8-9	Report numbers of outcome events or summary measures
Main results	9	(*a*) Give unadjusted estimates and, if applicable, confounder-adjusted estimates and their precision (eg, 95% confidence interval). Make clear which confounders were adjusted for and why they were included
		(*b*) Report category boundaries when continuous variables were categorized
		(*c*) If relevant, consider translating estimates of relative risk into absolute risk for a meaningful time period
Other analyses	10	Report other analyses done—eg analyses of subgroups and interactions, and sensitivity analyses
**Discussion**		
Key results	10-11	Summarise key results with reference to study objectives
Limitations	11	Discuss limitations of the study, taking into account sources of potential bias or imprecision. Discuss both direction and magnitude of any potential bias
Interpretation	12-15	Give a cautious overall interpretation of results considering objectives, limitations, multiplicity of analyses, results from similar studies, and other relevant evidence
Generalisability	15-16	Discuss the generalisability (external validity) of the study results
**Other information**		
Funding	19	Give the source of funding and the role of the funders for the present study and, if applicable, for the original study on which the present article is based
